# Maintenance Dialysis in a District General Hospital

**Published:** 1991-03

**Authors:** Richard A. Banks, Jackie Folley

**Affiliations:** Consultant Renal and General Physician, Gloucestershire Royal Hospital; Clinical Nurse, Gloucestershire Royal Hospital

## Abstract

A programme of Continuous Ambulatory Peritoneal Dialysis has been in progress at the Gloucester Royal Hospital since January 1988. After 2 years patient and technique survival was 81% and 73% respectively, very similar to that in established British Renal Units. Management of end stage renal failure at the local District General Hospital has meant that patients no longer have to travel long distances to the Regional Renal Unit.


### Maintenance Dialysis in a District General Hospital

**Richard A. Banks MD, FRCP**
Consultant Renal and General Physician, Gloucestershire Royal Hospital

**Jackie Folley SRN**
Clinical Nurse, Gloucestershire Royal Hospital

### SUMMARY

A programme of Continuous Ambulatory Peritoneal Dialysis has been in progress at the Gloucester Royal Hospital since January 1988. After 2 years patient and technique survival was 81% and 73% respectively, very similar to that in established British Renal Units. Management of end stage renal failure at the local District General Hospital has meant that patients no longer have to travel long distances to the Regional Renal Unit.

### INTRODUCTION

In the United Kingdom, the treatment of end stage renal failure by maintenance haemodialysis has been concentrated in large centres thus centralising technical expertise. With the introduction of Continuous Ambulatory Peritoneal Dialysis (C.A.P.D.), it was suggested that maintenance dialysis could be run from District general hospitals without the need for technical support.[1] This did not have universal support, a particular concern being the lack of acute haemodialysis back-up.[2] This paper describes the first 2 years of C.A.P.D. in a District general hospital.

### PATIENTS AND METHODS

Between February 1988 and March 1990, the Gloucester Royal Hospital accepted 24 new patients for C.A.P.D. training. Four patients were transferred from other units and over the same time period, 16 patients were referred elsewhere for maintenance haemodialysis. Ages of the 28 on C.A.P.D. ranged from 25 to 79 years. Nine were diabetic. In the weeks prior to starting, patients were invited to the ward to have C.A.P.D. demonstrated and preliminary training given. Abdominal (Tenckhoff) catheters were inserted under local anaesthetic by the nephrologist and where possible patients were sent home for 5 to 7 days before re-admission for training which then took a mean of 10 (range 7–12) days. Patients were always admitted to a single medical ward where 3 nurses (the ward sister and 2 S.E.N.'s) have particular C.A.P.D. training and responsibility but where all S.R.N.'s have C.A.P.D. experience ensuring that expertise is available at all times.

Peritonitis diagnosed clinically and by the presence of 100 white blood cells per microlitre of dialysis effluent,[8] occurred 32 times in 10 patients. Twenty-one episodes were confined to 3 patients all of whom have been transferred for maintenance haemodialyis. Eighteen patients never experienced peritonitis. Mean peritonitis rate was one per patient per 10.5 months, similar to that in many established `British renal units[5, 7] (see table). Treatment by various combinations of intravenous or intra-peritoneal vancomycin, intra-peritoneal gentamicin and oral ciprafloxacin was initiated by junior medical staff following a detailed protocol that also served as an aid for consultants covering the nephrologist when on leave. On 22 occasions patients were sent home for treatment of their peritonitis. Ten episodes required hospital admission for periods ranging from 1 to 7 days. In 3 patients relapse of peritonitis was treated by installation of urokinase or by catheter replacement in a single procedure. Although available at this hospital, temporary haemodialysis to "rest" the abdomen was not necessary.

Correspondence to: R.A. Banks, Gloucestershire Royal Hospital, Great Western Road, Gloucester.

Four patients died (1 empyema, 1 disseminated carcinoma and 2 sudden deaths). One patient was successfully transplanted and 1 regained renal function. The 2 year patient and technique survival was 81% and 73% respectively *(table)*. The cost of the programme was met from a budget previously used to fund direct charges from the Regional Renal Unit.

### COMMENTS

There are fewer renal units and nephrologists in the United Kingdom relative to population than most other European countries.[3] Acceptance rates have reflected this under-provision but have improved over the last few years,[4] increasing the pressure on the established units.

C.A.P.D. may provide an opportunity for nephrologists in District general hospitals to ease this pressure while at the same time giving a local service for the population and broadening the interest and experience of the nursing and junior medical staff. For 2 years we have offered a service to a catchment population on 550,000. Hitherto, patients with end stage renal failure in Gloucestershire needed to travel up to 60 miles to Bristol for renal replacement therapy. Our experience is that C.A.P.D. can be undertaken in a District general hospital by nurses without prolonged specialist renal training. Bed occupancy (excluding training time) has averaged 10 days per patient per year and has been minimised by starting outpatient training before commencing C.A.P.D., discharging patients for several days after Tenckhoff catheter insertion, predominantly outpatient management of peritonitis and catheter removal and replacement as a one-step procedure without the need for temporary haemodialysis. Our experiences suggests that only 2 extra beds need be found to sustain C.A.P.D. for a District of 300, 000 (see table).

Our results are encouraging. Peritonitis rates and survival compare well with large, established U.K. renal units.[5] Acceptance rates for renal replacement therapy vary inversely with the distance from a renal unit.[6] It is therefore not surprising that our annual acceptance rates have more than doubled from 20 to 44 per million population since the advent of C.A.P.D.

We believe that our experience illustrates that a C.A.P.D.. programme can be run by a district general hospital with the expertise of a nephrologist and the co-operation and dedication of general medical nursing staff. Immediate availability of acute

| | UK[5] Multicentre | Newcastle[7] | Gloucester |
|---|---|---|---|
| Patient Number | 610 | 229 | 28 |
| 2yr. Patient Survival % | 80 | 79 | 81 |
| 2yr. Technique Survival % | *75 | 66 | 73 |
| Peritonitis/Pat./Yr. | 1.34 | 1.4 | 1.1 |
| Peritonitis relapse % | 17 | 29 | 15 |
| Temporary Change To Haemodialysis | 233 pts. 7.6 days/ pat/yr. | 55 pats. 0–2 months | 0 |
| Hospital Admission Days/Pat./Yr. | 14.7 | 12.3 | 7.1 |
| Extra Beds/100 Pats. | 5 | 4 | 2 |

*Deaths excluded haemodialysis is not mandatory though the co-operation of the Regional renal unit is necessary. C.A.P.D. at a District general hospital is rewarding for the staff, beneficial to the local dialysis population and likely to increase new patient acceptance rates. Moreover, with the widespread introduction in April 1991 of direct charging of Districts by Regional Renal Units, a locally run maintenance dialysis program may be economically as well as professionally attractive.

### ACKNOWLEDGEMENTS
We warmly acknowledge the industry of the nursing, dietetic, clerical and secretarial staff that has contributed to the success of this venture.

### ADDENDUM
Since February 1990 seventeen additional patients have started C.A.P.D. One has needed temporary haemodialysis after pseudomonas peritonitis and we wish to thank Southmead Renal Unit for their co-operation.

### REFERENCES
1. GABRIEL R. (1982) Renal failure – dilemma and developments. *Br. Med. J.* **284,** 1406.
2. COLES G.A. (1985) Is peritoneal dialysis a good long term treatment? *Br. Med. J.* **290:** 1164–1166.
3. TUFRESON, G., GEERLINGS, W., BRUNNER, F.P. et al. (1989) Combined report on regular dialysis and transplantation in Europe XIX 1988. *Nephrol. Dial. Transplant* **4** (suppl. 4), 5–29.
4. WING, A.J., BROYER, M., BRUNNER, F.P. et al. (1988) Demography of dialysis and transplantation in Europe in 1985 and 1986: Trends over the previous decade. *Nephrol. Dial. Transplant.* **3,** 714–727.
5. GOKAL, R., JAKUBOWSKI, C., KING, J. et al. (1987) Outcome in patients on continuous ambulatory peritoneal dialysis and haemodialysis: 4 year analysis of a prospective multi-centre study. *Lancet* **2,** 1105–1108.
6. DALZIEL, M., GARRETT, C. (1987) Intraregional variation in treatment of end stage renal failure. *Br. Med. J.* **294,** 1382–3.
7. HEATON, A., RODGER, R.S.C., SELLARS, L. et al. (1986) Continuous Ambulatory Peritoneal Dialysis after the honeymoon. A review of experience in Newcastle 1979–84. *Br. Med. J.* **393,** 938–941.
8. Working Party of the British Society for Antimicrobial Chemotherapy (1987). Diagnosis and management of peritonitis in Continuous Ambulatory Peritoneal Dialysis. *Lancet,* **1,** 845–849.

